# Variation in three community features across habitat types and scales within a 15‐ha subtropical evergreen‐deciduous broadleaved mixed forest dynamics plot in China

**DOI:** 10.1002/ece3.4655

**Published:** 2018-11-11

**Authors:** Guang Feng, Jun‐Qing Li, Run‐Guo Zang, Yi Ding, Xun‐Ru Ai, Lan Yao

**Affiliations:** ^1^ Key Laboratory for Silviculture and Conservation of Ministry of Education Beijing Forestry University Beijing China; ^2^ Key Laboratory of Forest Ecology and Environment, State Forestry Administration, Institute of Forest Ecology, Environment and Protection Chinese Academy of Forestry Beijing China; ^3^ Co‐Innovation Center for Sustainable Forestry in Southern China Nanjing Forestry University Nanjing Jiangsu China; ^4^ School of Forestry and Horticulture Hubei University for Nationalities Enshi Hubei China

**Keywords:** evergreen and deciduous broadleaved mixed forests, habitat classification, multiscale analysis, plant diversity and forest structure, variation partition

## Abstract

The evergreen and deciduous broadleaved mixed forests (EDBMFs) belong to one of the ecosystems most sensitive to environmental change, however, little is known about the environmental determinants for their plant diversity and forest structure at different habitat types and spatial scales. Here, we used data from a 15‐ha (300 × 500 m) forest dynamic plot (FDP) of an old‐growth EDBMF to examine the patterns and determinants of the three community features (stem abundance, rarefied species richness and basal area [BA]) in three habitat types (ridge, hillside and foothill) and at three spatial scales (20 × 20 m, 50 × 50 m, and 100 × 100 m). We found that the three community features significantly changed with habitat type, but only one of them (rarefied richness) changed with scale. Among spatial scales, the principle environmental factors that widely affected community features were pH, soil organic matter, and total phosphorus, while these effects only taken place at certain habitat. Variations in the three community features explained by soil conditions were generally greater than those explained by topographical conditions. With changes in habitat type, the proportion of variations explained by environmental conditions was 31%–53%, 8%–25%, and 18%–26% for abundance, rarefied richness, and BA, respectively. With increasing spatial scale, the variations explained by environmental conditions were 44%–75% for abundance, 28%–95% for rarefied richness, and 18%–86% for BA. Our study demonstrated that environmental factors had great impacts on the plant diversity and forest structure in the EDBMFs, especially the soil factors such as pH. In addition, the importance of the environmental determinants on these community features was highly related to the spatial scale.

## 1 INTRODUCTION

Environmental conditions ubiquitously varying in terrestrial ecosystems involve various processes in affecting community patterns (Chisholm et al., 2014; García‐Palacios, Maestre, Bardgett, & Kroon, 2012; Laanisto et al., 2013; Shmida & Ellner, 1984). Ecologically, strong environmental heterogeneity can facilitate the coexistence of species with different habitat requirements, or enhance persistence by sheltering plants from adverse environmental conditions and competition (Stein, Gerstner, & Kreft, 2014; Tews et al., 2004). Moreover, habitat fragmentation in extreme fine‐scale heterogeneity potentially breaks dynamic equilibrium between immigration and extinction, and leads to ambiguous community patterns (Laanisto et al., 2013). From an evolutionary aspect, environmental heterogeneity has been argued to promote diversification through isolation and adaptation (Kallimanis et al., 2010; Simpson, 1964). Thus, regions with strong topographic heterogeneity containing physical obstacles and isolated valleys or peaks, coupled with other abiotic variations for physiological barriers, limited gene flow and were associated with specialization and adaptive radiation via a wider variety of environmental pressures and opportunities (Stein et al., 2014). In mountainous areas, soil and topographic conditions often show strong variability, even co‐variability (Enoki, Kawaguchi, & Iwatsubo, 1996). Soil and topographical factors have strong influences on plant performance (García‐Palacios et al., 2012; John et al., 2007), and other landscape heterogeneity (Legendre et al., 2009; Punchi‐Manage et al., 2014). Hence, it is imperative to synchronously consider soil and topographic variables and distinguish their effects to gain comprehensive insights on the importance of environmental determinants on plant diversity and forest structure.

In mountain systems, the impact of topographic heterogeneity has been partly attributed to high rates of shifts in habitat types over relatively short distances (Ruggiero & Hawkins, 2008; Stein et al., 2014). On this basis, several attempts to directly classify habitat types in large forest dynamic plots (FDPs) have resulted in great achievements in understanding the local habitat association of natural communities. Using torus‐translation tests, Gunatilleke et al. (2006) and Harms, Condit, Hubbell, and Foster (2001) found 80% and 64% of tested species in Sinharaja and Barro Colorado Island (BCI) plots, respectively, had significant associations to at least one habitat, indicating the universality of habitat association for most species. In a Gutianshan plot, Lai, Mi, Ren, and Ma (2009) found that more species at the sapling and juvenile stages occurred at ridges, while more species of adult tree occurred at valleys, suggesting that the habitat preference of a plant is related to its life stage. All of these habitat classifications are based on topography, which are widely considered as integrated variables for proxies of light, moisture, nutrient, and thermal conditions (Baldeck et al., 2013; Legendre et al., 2009; Punchi‐Manage et al., 2014). Hence, it is reasonable to hypothesize that plant diversity and forest structure will respond to habitat change because of multiple shifts in environmental conditions.

Meanwhile, the importance of scale is a core tenet of the ecological sciences (Schneider, 2001). A growing recognition of scale focuses on the fact that ecological patterns should be variously read at different spatial scales, as underlying processes are not independent of scale. For instance, in a single species, processes such as competition and dispersal often operate at different spatial scales (Willis & Whittaker, 2002). The roles of topographic heterogeneity in affecting species turnover and promoting allopatric or ecological speciation are also reflected at different scales (Stein et al., 2014). In recent decades, several large FDPs have been established with standard sampling protocols (Anderson‐Teixeira et al., 2015); examining these FDPs at multiple scales has allowed ecologists to achieve great understanding of these scale‐dependent patterns and their underlying processes. For instance, by examining 25 FDPs worldwide, Chisholm et al. (2013) observed a trend of positive correlations between richness and ecosystem functions that occurred at only small scales, and they partially attributed this outcome to the scale‐dependent sampling effect. In Gutianshan, BCI, and Sinharaja plots, ecologists revealed an enhanced role of topographical filtering with increasing scale (Hu, Jin, Liu, & Yu, 2014; Kanagaraj, Wiegand, Comita, & Huth, 2011; Legendre et al., 2009; Punchi‐Manage et al., 2013). In this context, large stem‐mapped FDPs, which provide valuable data sources for understanding the mechanism behind variations in local plant diversity and forest structure (Condit et al., 2006), should be accepted as ideal settings for assessing scale effects.

Evergreen and deciduous broadleaved mixed forests (EDBMFs), the zonal climax vegetation type in northern subtropical and mid‐subtropical mountainous regions (Myers, Mittermeier, Mittermeier, Da, & Kent, 2000), is one of the ecosystems most sensitive to environmental changes (Ge & Xie, 2017; Myers et al., 2000; Seddon, Macias‐Fauria, Long, Benz, & Willis, 2016). Given their high biodiversity and small geographical range in the world, understanding the environmental determinants of EDBMFs is an essential step for further revealing what maintains the species diversity of this vegetation type. At regional scale, latitude‐associated minimum temperature and mean annual precipitation were confirmed to contribute to the vegetation patterns of subtropical EDBMFs by affecting species compositions and the relative dominance of evergreen and deciduous (Ge & Xie, 2017). At local scale (within a climate zone), it is noteworthy that both microclimate and soil are habitat‐associated, and evidences support their effects on the coexistence of evergreen and deciduous tree species and the biomass, diversity and species composition of EDBMFs (Fang et al., 2016; Huang et al., 2015; Song, Kohyama, & Da, 2014; Xu et al., 2015). The fundamental cause for such a variety of environmental factors exerting impacts on EDBMFs is the significant niche differences among diverse species there, especially for evergreen and deciduous species with different leaf habits, which have long been deemed to relate to distinct strategies for dealing with environmental conditions (Ge & Xie, 2017; van Ommen Kloeke, Douma, Ordoñez, Reich, & Bodegom, 2012). Deciduous species use an opportunist strategy to maximize the photosynthetic rate during a favorable period and to minimize transpiration and respiration (via shedding leaves) to reduce costs during seasonal drought or low temperatures, while evergreens adopt a conservative strategy to maintain a long photosynthetic period by developing tough leaves to withstand unfavorable conditions (Givnish, 2002; Reich, Walters, & Ellsworth, 1992; Villar, 2001). This type of disparity in trade‐off results in distinct nutrient requirements and habitat preferences between evergreen and deciduous species (Aerts & Chapin, 2000; Givnish, 2002). Therefore, an essential mechanism accounting for the community patterns of EDBMFs can be expected as the environmental determinant, especially when these forests spread over mountainous areas where heterogeneous landscapes maintain diverse habitats. However, the importance of environmental determinants for the plant diversity and forest structure of EDBMFs, as well as the relative role of soil and topographical conditions, has seldom been examined and quantified across habitats and scales.

Here, we integrate the topographical and soil variables in a 15‐ha FDP of old‐growth EDBMFs located in the Mulinzi National Nature Reserve, Central China, to examine the patterns and environmental determinants of three community features (stem abundance, rarefied species richness, and basal area [BA]) across three habitat types (ridge, hillside, and foothill) and three spatial scales (20 × 20, 50 × 50, and 100 × 100 m). The general objectives of this study were to examine (a) the variations in these community features among habitat types and spatial scales; (b) how many variations in these community features can be explained by environmental conditions at different habitat types and spatial scales; and (c) which environmental factor play the foremost role, as well as the relative importance of soil and topographical conditions, in determining the three community features.

## 2 MATERIALS AND METHODS

### 2.1 Study area

**Figure 1:**
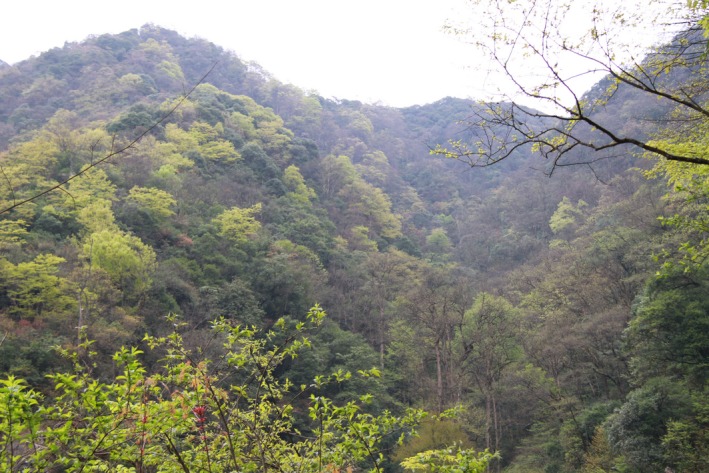
Photograph of the old‐growth evergreen and deciduous broadleaved mixed forests in our study area (Mulinzi Reserve, Central China). It was taken after some deciduous plants started growing leaves in spring

Our study area was located in the Mulinzi (MLZ) National Nature Reserve, southwest Hubei Province, Central China (29°55′59″–30°10′47″N, 109°59′30″–110°17′58″E). The mean annual temperature of this area is ~15.5°C, and the annual effective accumulated temperature (≥10°C) is approximately 4,925.4°C. The mean annual air relative humidity (under canopy) is ~90% and the annual precipitation ranges from 1,700 to 1,900 mm. In the core zone of this reserve, a vast area of continuous old‐growth EDBMF exists (Figure 1), which is where we established a 15‐ha FDP (300 × 500 m; 30°4′28.50″N, 110°12′19.30″E) according to the standard of the Center of Tropical Forest Science (Condit, 1998).

This plot embodied an old‐growth EDBMF without record of anthropogenic disturbance since the 1920s. The first census was finished in 2014, and all woody plants with a diameter at breast height (dbh) ≥1 cm were tagged, mapped, measured, and identified. In total, there were 84,189 individuals, comprising 227 species (71 species of evergreen tree species and 156 species of deciduous tree species), 118 genera and 57 families (Yao, 2016).

### 2.2 Measurement of environmental variables

Elevation data were recorded at four corners of each 20 × 20 m quadrat using real‐time kinematic method. Three topographical variables were measured: mean elevation, slope, and convexity. The MLZ plot was further divided into 20 × 20 m (small scale), 50 × 50 m (intermediate scale), and 100 × 100 m (large scale) subplots. In each subplot, the mean elevation was calculated as the mean value of four corner elevations. Slope was then calculated as the average deviation angle from four planes (sequentially generated by taking elevation at three corners) to horizontal. Convexity was determined as the mean elevation of the focal quadrat minus the mean elevation of surrounding neighbors.

In 2014, we sampled soils at three points in each 20 × 20 m quadrat (one at the center and two at the diagonal locations) and mixed them together after removing litter and humus layers from the top level of soil. The soil samples were air‐dried and transported to the soil laboratory for chemical analysis, including the determination of six soil properties: pH, soil organic matter (SOM), total nitrogen (TN), total phosphorus (TP), available nitrogen (AN), and available phosphorus (AP). Environmental data of 50 × 50 and 100 × 100 m subplots were calculated as the averages of the values inside 10 × 10 and 20 × 20 m subplots, respectively; data of 10 × 10 m subplots were measured by kriging interpolation.

### 2.3 Statistics

We focused on three community features of plant diversity and forest structure. Specifically, we used abundance and BA as measure of forest structure, and rarefied richness as a measure of plant diversity. The three community features were calculated at subplots of different scales. Rarefied richness, the expected number of species in random samples from the community, was mathematically obtained from species‐abundance curves by uniformly sampling 100 individuals at subplots of different scales. For those plots (*n* = 25) without enough number of individuals, we used species richness instead of rarefied richness.

The habitat types were categorized using a classification of complete linkage agglomerative clustering of topographical data (Supporting Information Figure S1). One‐way ANOVA and Tukey's honest significant difference test (Tukey‐HSD) were used to examine whether plant diversity and forest structure at different habitats and scales were significantly different.

To quantify how many variations in community features among habitats and scales were explained by different groups of environmental conditions (soil and topographical conditions) and realizing that their coupling with spatial autocorrelation of the three community features could not be neglected (Supporting information Figure S2), we used variation partitioning based on a simultaneous autoregressive (SAR) model of the error term (Coyle & Hurlbert, 2016; Özkan, Svenning, & Jeppesen, 2013). Ordinary least squares (OLS) models including all environmental factors were first fitted and optimized by stepwise selection, and then the final formulas were refitted by the SAR models (Dorman et al., 2007; Özkan et al., 2013; Stein et al., 2015). Variation partition analysis measured the pseudo‐*R* ^2^ values (Nagelkerke, 1991; Özkan et al., 2013) of the full model that included all variables in the final formula (left by stepwise selection) and the partial models that separately included only soil variables or topographical variables in the final formula. In the SAR model, we specified the row‐standardized coding as a weighting scheme for the spatial matrix, and eight neighbors (i.e., on the grid map, these grids are mostly connected or close to the focal site at all possible directions) as a neighborhood for representing the core influence zone of spatial autocorrelation. By doing so, the spatial autocorrelations in residuals were reduced to negligible levels (Supporting Information Figure S2).

However, the models used in variation partitioning, rather focusing on the explanatory power of certain environmental groups, were not appropriate to be used for measuring the contribution of each factor. Meanwhile, environmental conditions, especially topographical conditions, can play a role in community features by affecting soil conditions, but soil and topographical conditions cannot achieve that by affecting another topographical variable. Then, we separately examined the contributions of soil and topographical conditions to the community features at different habitats and scales. Specifically, based on the SAR models, formulas including only soil variables were used to examine the contribution of each soil factor, while models including one topographical factor in addition to soil variables (used as the control variables) at each modeling to analyze the contribution of each topographical factor.

For each of the community features, we used the Spearman rank correlation test to examine whether its pattern at one scale was consistent with that at other scales. Given that different spatial scales (grain sizes) did not have an identical number of subplots, we divided the patterns of three community features of different scales into the same number of subplots for a one‐to‐one match (He, Lafrankie, & Song, 2002). Specifically, those community patterns at different scales were uniformly divided into 10 × 10 m patterns that resulted in 1,500 subplots for site‐to‐site correlation.

All procedures were implemented in R software (v.2.9.2; R Core Team, 2014). Tests of SAR models were conducted using “spdep” package (Bivand, 2015), and other operations were performed in the “stats,” “vegan,” “gstat,” and “ncf” packages (Oksanen et al., 2009).

## 3 RESULTS

### 3.1 Habitat classification

**Figure 2:**
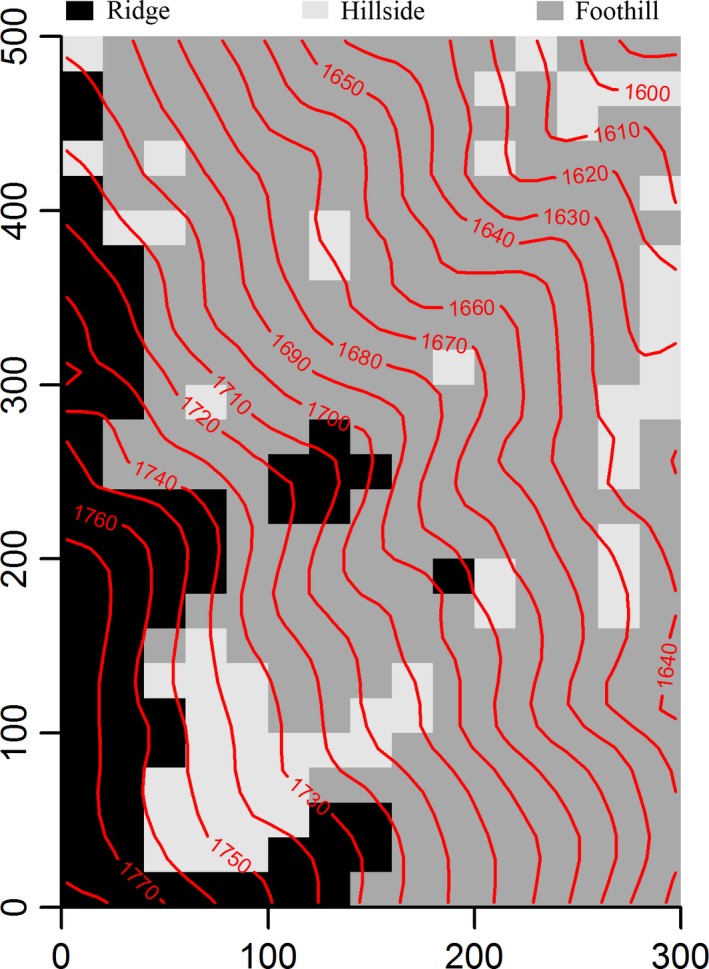
Distribution map of the three habitat types in the MLZ plot. Red lines are contours with intervals of 10 m

Elevation in the MLZ plot ranged from 1,583.4 m to 1,785.2 m (Figure 2). Complete linkage agglomerative classification identified three topographical habitats (Supporting Information Figure S2): ridges (*n* = 66), hillsides (*n* = 55), and foothills (*n* = 254). Except for SOM, soil variables significantly varied among three habitats (ANOVA, *p < *0.05; Supporting Information Figure S3). From ridges to foothills, the pH value significantly decreased, while the soil nutrient contents increased (Tukey‐HSD, *p < *0.001).

### 3.2 Variations in community features among habitat types and spatial scales

**Figure 3:**
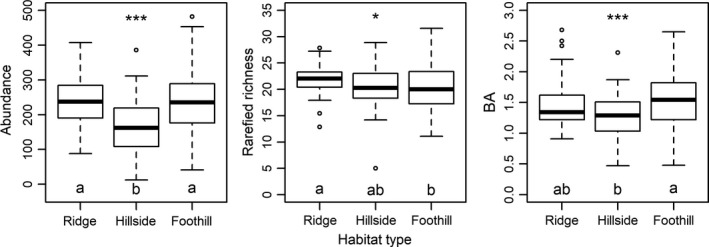
Variations in plant diversity and forest structure among three habitats. Habitats labeled with identical letters have nonsignificant differences in their values of community feature (Tukey‐HSD, *p* > 0.05), and habitats labeled with different letters have significant differences in their values of community feature (*p < *0.05). *p < 0.05; **p < 0.01; ***p < 0.001 by one‐way ANOVA

Abundance, rarefied richness, and BA significantly varied among habitats (ANOVA, *p < *0.05; Figure 3). The abundance and BA had their lowest values at hillside, while their values at ridge and foothill were not significantly different (Tukey‐HSD, *p < *0.05); rarefied richness varied significantly among habitats, decreasing from ridge to foothill.

**Figure 4:**
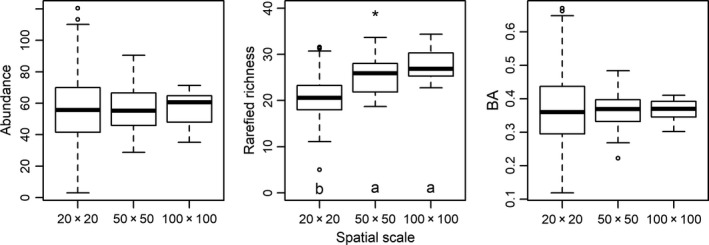
Variations in plant diversity and forest structure among three scales. Since basal area (BA) and abundance can accumulate with increasing area, their patterns at different scales are calculated as the average values of BA and abundance per 100 m^2^ area. Scales labeled with identical letters have nonsignificant differences in their values of community feature (Tukey‐HSD, *p* > 0.05), and scales labeled with different letters have significant differences in their values of community feature (*p* < 0.05). *p < 0.05; **p < 0.01; ***p < 0.001 by one‐way ANOVA

Rarefied richness significantly varied among spatial scales (ANOVA, *p* < 0.001; Figure 4), but abundance and BA did not. Rarefied richness significantly increased from small to intermediate scale (Tukey‐HSD, *p* < 0.05), while it did not vary significantly between intermediate and large scale (*p* > 0.05). Community features at different scales showed significant positive correlations (Spearman rank correlation test, *p* < 0.001; Table 1).

**Table 1:** Correlations between community features at different scales

Scale	Abundance		Rarefied richness		BA	
	20 × 20	50 × 50	20 × 20	50 × 50	20 × 20	50 × 50
50 × 50	0.64***		0.65***		0.40***	
100 × 100	0.51***	0.74***	0.55***	0.77***	0.31***	0.57***

Notes

The 100 × 100 m (*n* = 15), 50 × 50 m (*n* = 60), and 20 × 20 m (*n* = 375) patterns of community features were uniformly divided into 10 × 10 m patterns (*n* = 1,500) for correlations of site‐to‐site match.

BA: basal area.

*p* < 0.05.

*p* < 0.01.

a *p* < 0.001.

### 3.3 Effect size of different environmental factors

The principle environmental factor that had the largest standard effect size for community features among spatial scales was mostly one of the following soil factors (Table 2): pH, SOM, and TP. These three soil factors also had constant effects on community features across scales: pH had significant negative effects on BA and positive effects on rarefied richness, while SOM and TP had significant effects on abundance. Slope was the principle topographical factor that had significant effects on community features at small and large scales. The other environmental factors had few effects on community features at small scale, but they had significant effects on those at intermediate or large scales.

**Table 2:** The influences of environmental factors on community features in three habitat types

Variables	Ridge			Hillside			Foothill		
	Abundance	BA	Rarefied richness	Abundance	BA	Rarefied richness	Abundance	BA	Rarefied richness
pH	1.32	−1.03	1.87	−1.33	−3.82***	2.12*	−3.00**	−5.06***	3.13**
SOM	3.96***	0.60	−0.34	2.05*	1.54	0.18	1.53	0.03	−0.88
TP	0.01	1.14	0.94	−5.18***	−0.9	−1.33	−4.10***	−3.69***	−0.51
AN	−2.44*	−0.42	−0.34	−1.32	−0.05	0.26	0.85	2.33*	−0.36
AP	1.95	0.91	−1.35	3.39***	0.04	0.59	0.32	0.23	−0.75
TN	−0.34	0.72	−1.06	1.32	0.37	0.05	−0.48	−1.09	0.23
Elevation	1.11	−1.83	0.43	−2.53*	0.68	0.50	−1.39	0.73	−0.25
Convexity	1.10	−0.82	0.20	−1.87	0.49	−0.79	1.05	2.08*	−1.12
Slope	1.67	0.61	0.10	−0.66	−0.54	0.25	3.13**	2.24*	0.82

Values shown in this table are the standard effect sizes.

AN: available nitrogen; AP: available phosphorus; BA, basal area; SOM: soil organic matter; TN: total nitrogen; TP: total phosphorus.

a *p* < 0.05.

b *p* < 0.01.

c *p* < 0.001.

The principle factors that had relatively wide influences on community features among habitat types were also pH, SOM, and TP (Table 3). Their effects among habitats were the same as those among scales, but none of their effects kept constant across habitats. The effects of pH and TP only taken place at hillside and foothill, while the effects of SOM taken place at ridge and hillside. The other environmental factors had few effects on community features among habitats.

**Table 3:** The influences of environmental factors on community features at three spatial scales

Variables	20 × 20 m			50 × 50 m			100 × 100 m		
	Abundance	BA	Rarefied richness	Abundance	BA	Rarefied richness	Abundance	BA	Rarefied richness
pH	−3.34***	−6.70***	2.52*	−0.50	−8.84***	7.20***	1.18	−6.60***	7.66***
SOM	2.31*	0.52	−2.77**	2.77**	−1.88	1.10	3.80***	1.49	−1.87
TP	−5.28***	−3.83***	−0.05	−2.36*	−0.56	−0.58	−2.67**	2.33*	−2.36*
AN	−0.13	1.88	1.73	0.29	−0.29	−2.02*	−1.13	−2.82**	0.84
AP	1.91	0.51	0.02	0.72	−2.36*	2.80**	−1.76	−2.80**	3.02**
TN	0.49	−0.54	−0.11	−0.42	−0.57	1.62	4.43***	1.06	1.01
Elevation	−0.70	0.13	0.89	−1.58	−0.40	0.81	2.60**	1.61	5.82***
Convexity	1.63	1.86	−0.98	−1.16	−0.91	0.98	2.28*	1.42	6.00***
Slope	5.14***	3.66***	0.99	−0.81	−1.00	−1.23	2.65**	2.03*	4.00***

Values shown in this table are the standard effect sizes.

AN: available nitrogen; AP: available phosphorus; BA: basal area; SOM: soil organic matter; TN: total nitrogen; TP: total phosphorus.

a *p* < 0.05.

b *p* < 0.01.

c *p* < 0.001.

### 3.4 Variations in community features explained by environmental conditions

The explained variations in the three community features varied with habitat type (Table 4). The proportion of explained abundance variations ranged from 31% at foothill to 53% at hillside, that of rarefied richness ranged from 8% at hillside to 25% at foothill, and that of BA ranged from 18% at foothill to 26% at ridge. In each habitat type, the variations explained by topographical conditions could also be mostly explained by soil conditions as shared effects, while soil conditions totally explained more variations in community features than topographical conditions.

**Table 4:** Variation partitioning in three habitat types

Habitat	Formulas used in the SAR models	Explained fractions (%)					
		(a)	(b)	(c)	(d)	(e)	(f)
Ridge	Abundance ~ AP + pH + SOM + AN + Slope + Convex	53	47	41	35	12	6
	Rarefied richness ~ pH + TN	10	10	0	0	10	0
	BA ~ pH + TP + Elevation	26	23	21	18	5	3
Hillside	Abundance ~ AP + TP + SOM + AN + Elevation	52	43	30	21	22	9
	Rarefied richness ~ pH	8	8	0	0	8	0
	BA ~ pH + SOM	25	25	0	0	25	0
Foothill	Abundance ~ pH + TP + SOM + Slope + Elevation	31	28	24	21	7	3
	Rarefied richness ~ pH + AN	25	25	0	0	25	0
	BA ~ pH + TP + AN + Slope + Convex	18	13	11	6	7	5

Ordinary least squares models including all environmental factors were first fitted and optimized by stepwise selection, and then these formulas were used to fit the SAR models. Fractions (a–c) stand for the proportion of variations explained by all environmental factors, soil factors and topographical factors in the left formulas, respectively; (d) represent those explained by the shared effects of topographical and soil conditions; (e, f) stand for those explained by the pure effect of soil and topographical conditions, respectively.

AN: available nitrogen; AP: available phosphorus; BA: basal area; SOM: soil organic matter; TN: total nitrogen; TP: total phosphorus.

More variations in community features were explained by environmental conditions at a larger spatial scale (Table 5). With increasing scale, the explained variations increased from 44% to 75% for abundance, 28% to 95% for rarefied richness, and 18% to 86% for BA. It was also noteworthy that, at large scale, pH solely accounted for 71% of variations in BA and 83% of variations in rarefied richness (Supporting Information Figure S5). Generally, soil variables explained more variations in community features than topographical variables, especially at large scale.

**Table 5:** Variation partitioning at three spatial scales

Scale	Formulas used in the SAR models	Explained fractions (%)					
		(a)	(b)	(c)	(d)	(e)	(f)
20 × 20 m	Abundance ~ AP + TP + SOM + AN + Slope + Elevation + Convex	44	37	42	35	2	7
	Rarefied richness ~ pH + TP + AN + Convex	28	28	26	26	2	0
	BA ~ pH + TP + AN + Slope + Convex	18	13	8	3	10	5
50 × 50 m	Abundance ~ TP + SOM + Elevation	45	43	28	26	17	2
	Rarefied richness ~ pH + AN + AP + Slope	51	50	33	32	18	1
	BA ~ AP + pH + TP	40	40	0	0	40	0
100 × 100 m	Abundance ~ TN + AP + AN + SOM + Slope	75	64	14	3	61	11
	Rarefied richness ~ TN + AP + TP + pH + Convex	95	89	50	44	45	6
	BA ~ AP + pH + Slope + Elevation	86	82	47	43	39	4

Note

Ordinary least squares models including all environmental factors were first fitted and optimized by stepwise selection, and then these formulas were used to fit the SAR models. Fractions (a–c) stand for the proportion of variations explained by all environmental factors, soil factors and topographical factors in the left formulas, respectively; (d) represents those explained by the shared effects of topographical and soil conditions; (e, f) stand for those explained by the pure effect of soil and topographical conditions, respectively.

AN: available nitrogen; AP: available phosphorus; BA: basal area; SOM: soil organic matter; TN: total nitrogen; TP: total phosphorus.

In addition, redundancy analysis (RDA) showed that the three community features varied significantly in their species compositions along most environmental resource axes (*p* < 0.05; Supporting Information Figure S6) and the compound environmental gradients (i.e., RDA axes; *p* < 0.001).

## 4 DISCUSSION

### 4.1 Community features change with habitat type

The EDMBFs are sensitive to climate conditions at regional scale (Ge & Xie, 2017; Givnish, 2002; Kröber, Heklau, & Bruelheide, 2015), while the key environmental conditions in affecting these forests at local scale may be either soil or topographical conditions (Fang et al., 2016; Huang et al., 2015; Song et al., 2014; Xu et al., 2015). In this study, we found that changes in habitat followed by shifts in soil conditions (Supporting Information Figure S3) led to substantial variations in community features among habitats (Figure 3), indicating that habitat‐associated soil conditions were the major factors in determining the plant diversity and forest structure of the EDBMFs at local scale. The highest rarefied richness and abundance were found at ridge and the largest BA was found at foothill. The principle soil factor in accounting for those variations among habitat types was pH, a habitat‐associated soil factor, while the principle topographical factor was slope (Table 3), reflecting mechanisms that related to physiological harshness (Gough, Shaver, Carroll, Royer, & Laundre, 2000) and within‐site environmental heterogeneity (Stein et al., 2014).

These outcomes supported the profound rules that high plant diversity and complex forest structure benefited from the less acidic soils (Chytrý et al., 2007; Schuster & Diekmann, 2003) and higher environmental heterogeneity (Hauffe, Schultheiß, Bocxlaer, Prömmel, & Albrecht, 2014; Redon, Bergès, Cordonnier, & Luque, 2014; Stein et al., 2014). Soils in the subplots of the MLZ plot were almost acidic (pH <5.; Supporting Information Figure S3). In acidic soils, a slight decrease in pH is often followed by a reduction in the availability of some plant nutrients, and the enhancements of solubility and availability of some cations (e.g., Al, Cu, Mn, and Zn) for plant uptake (Brady & Weil, 2004; John et al., 2007). However, in this plot, topographical conditions are the main causes of variations in soil nutrients, rather than pH, because physical erosion (e.g., overland flow and soil creep) and hydrologic leaching processes depending on the landscape surface can influence soil conditions (Chadwick & Asner, 2016). In addition, pH can have more direct influences on vegetation patterns because in soils that are too acidic, there is high Al toxicity and high hydrogen ion concentrations that hinder plant growth and survival (Chytrý et al., 2007; John et al., 2007; Schuster & Diekmann, 2003). Quite a few plant species fail to physiologically tolerate the acidic conditions will be excluded. For instance, a massive decrease in deciduous species along the ridge‐hillside‐foothill gradient (Supporting Information Figure S4) might be the outcome of increasing soil acidity, as it was well known that, unlike evergreen species with tolerance and even preference on acidic sites, deciduous species have poor tolerance on those conditions (Givnish, 2002; Monk, 1966). Given deciduous species contributed to the main part of species richness at all habitats, such decrease led to a reduction in diversity.

However, our result that habitat (foothill) with low diversity supports larger amount of BA than those with high diversity (Figure 3) is contrary to the general understanding that low diversity corresponds to low community productivity and less biomass through sampling effect and niche complementarity at small scale (Chisholm et al., 2013). The accumulation of BA is also sensitive to competition, especially for deciduous plants that had massive resource requirements for renewing leaves and maximizing photosynthesis and growth rate (Givnish, 2002; Monk, 1966). We argue that competition can be alleviated at foothill due to two possible reasons. First, after those deciduous plants with poor tolerance under acidic conditions are massively excluded, the remaining deciduous individuals (with certain tolerance) encounter each other less frequently at neighborhood, thus compete less. In other words, the biotic filtering (i.e., competition) may be less intensive when the abiotic filtering plays an important role in deciduous plants. This made the BA of deciduous to be less sensitive to habitat change than their richness and abundance (Supporting Information Figure S4). Second, slope is the key factor causing the different BA patterns between two habitats (hillside and foothill) with similar soil acidity and nutrient contents. Sites having steep slopes supported more amount of BA than those flat sites. Because slope is a measure of environmental heterogeneity (i.e., elevation decrease within the site), which has the ability to alleviate plant competition and facilitate species coexistence (Stein et al., 2014). Therefore, at foothill, evergreens with habitat preferences to such acidic condition accumulate large amount of BA, while deciduous species exhibit their potential of fast growing in this resource‐rich condition.

### 4.2 Community features change with spatial scale

Rarefied richness increasing with scale (Figure 4) demonstrated that the effect of the environmental heterogeneity was inherently linked with the area (Allouche, Kalyuzhny, Moreno‐Rueda, Pizarro, & Kadmon, 2012; Kadmon & Allouche, 2007). Abundance and BA did not change with scale because they could be simply added when scaling up. Generally, larger area scales are more likely to comprise a large number of habitat types or broad gradients in environmental conditions, which makes it difficult to distinguish the individual effects of scale or environmental heterogeneity (Ricklefs & Lovette, 1999). Meanwhile, large grains encompassing several habitat types offer more potential niches for the coexistence of species with diversified requirements, making it less possible when all niches are occupied. Sufficient number of unsaturated niches may result in low extinction rates, as Macarthur (1972) proposed that extinction rates rise abruptly as soon as all habitats are occupied by corresponding species. Stein and Kreft (2015) further interpreted Macarthur's view as heterogeneity creating shelters for population persistence and reducing stochastic extinction.

Plant diversity and forest structure often show inconsistency among scales, because noise or scale‐dependent processes might vary differently across scale (Wang et al., 2008; Weiher & Howe, 2003). For instance, He et al. (2002) found that community patterns among scales appeared to be independent in tropical rain forest and attributed this independence to the large small‐scale variations that probably arose from negative density dependence or other proximal neighborhood spacing processes. However, our study revealed strong self‐similarity of plant diversity and forest structure across scales (i.e., positive correlations among patterns at different scales; Table 1), indicating that small‐scale variations in community features were not very large. The drivers for the large variations at small scale might be less important in this study, because we found the important roles of environmental determinants at different scales, especially for the effects of pH, SOM, and TP that remained constant across scales (Table 3). In addition, rarefied richness had the strongest self‐similarity because small‐scale richness might also be controlled by the local realized species pool (Dufour, Gadallah, Wagner, Guisan, & Buttler, 2006; Weiher & Howe, 2003).

### 4.3 Environmental determinants change with habitat and scale

More variations in community features were explained by environment conditions at large scale (Table 5), especially that pH alone could explain the most variations in rarefied richness and BA (Supporting Information Figure S5), supporting an enhanced role of environmental determinants with increasing spatial scale (Shipley, Paine, & Baraloto, 2012). Meanwhile, although the explained variations also differed among habitats, such differences were smaller than those among spatial scales, indicating that the importance of environmental determinants on community features depended highly on spatial scale.

Scaling up changes the observed pattern of species coexistence from the individual level to community‐wide level (Punchi‐Manage et al., 2013), and the importance of environmental filtering relative to other processes (Tamme, Hiiesalu, Laanisto, Szavakovats, & Pärtel, 2010). A previous study in the MLZ plot revealed that half (50.9%) of the species were very rare and most dominant species were spatially aggregated (Yao, 2016), reflecting the best adapted species not being able to colonize available sites because of dispersal limitation that may invalidate the environmental filtering at a small scale. For example, the mass effects allow species to migrate in nearby unsuitable conditions beyond the limits of their ecological niches (Kunin, 1998; Palmer, Earls, Hoagland, White, & Wohlgemuth, 2010; Ron, Fragman‐Sapir, & Kadmon, 2017). In addition, stochastic extinction and biotic processes such as competition, which mostly take place at neighborhood scale, are considered as important drivers on small‐scale variations in community patterns (Baldeck et al., 2013; Punchi‐Manage et al., 2013). These processes may be more crucial than environmental filtering at small scale.

Even if the explained variations in community features did not massively change with habitat, factors determining community features changed with habitat (Table 2), especially those factors widely affecting community features across scales only shown influences at certain habitat. For example, soil pH affected the three community features at small scale, but these effects were limited to take place at foothill or hillside. We further found that soils at foothill and hillside are more acidic than those at ridge, and the pH ranges in ridge and hillside were similar (Supporting Information Figure S3). These outcomes demonstrate that, only in extremely acidic soils, a variety of pH values will influence the community features in the EDBMF, while in moderate acidic soils, other drivers such as those small‐scale processes mentioned above may account for community features.

### 4.4 Relative effects of topographical and soil conditions

An important finding in this study was that soil factors had greater effects on plant diversity and forest structure than topographic factors. Specifically, pH, SOM, and TP were the environmental factors that had relatively wide influences on community features among habitats (Table 2) and scales (Table 3), and their effect sizes were also very large. Moreover, soil conditions together explained more variations in community features than topographical conditions (Table 4 and 5). These results support the great importance of soil factors in determining the plant diversity and forest structure of the EDMBFs at local scale (Huang et al., 2015). Topographical conditions in the MLZ plot mainly impacted community features by determining the soil conditions, especially at small scale, where their effects are mostly shared. Consistent with other studies (Baldeck et al., 2013; Chadwick & Asner, 2016; Legendre et al., 2009), this finding highlights that topographical conditions are well proxies for soil conditions at local scale.

## CONFLICT OF INTEREST

None declared.

## AUTHOR CONTRIBUTION

Run‐Guo Zang and Jun‐Qing Li designed this study and provided theoretical guidance; Guang Feng performed field works, analyzed data, and wrote the manuscript; Yi Ding gave assistance in data analysis and language editing; Xun‐Ru Ai and Lan Yao formulated guidelines on fieldwork and offered convenient transportation.

## DATA ACCESSIBILITY

The research data has been deposited and opened in Dryad Digital Repository (https://doi.org/10.5061/dryad.s3np654).
