# Supplementary figures and images for: Variation in three community features across habitat types and scales within a 15‐ha subtropical evergreen‐deciduous broadleaved mixed forest dynamics plot in China
Source: Ecol Evol. 2018 Nov 11;8(23):11987–98. doi: 10.1002/ece3.4655 (PMC6303768; doi:10.1002/ece3.4655)

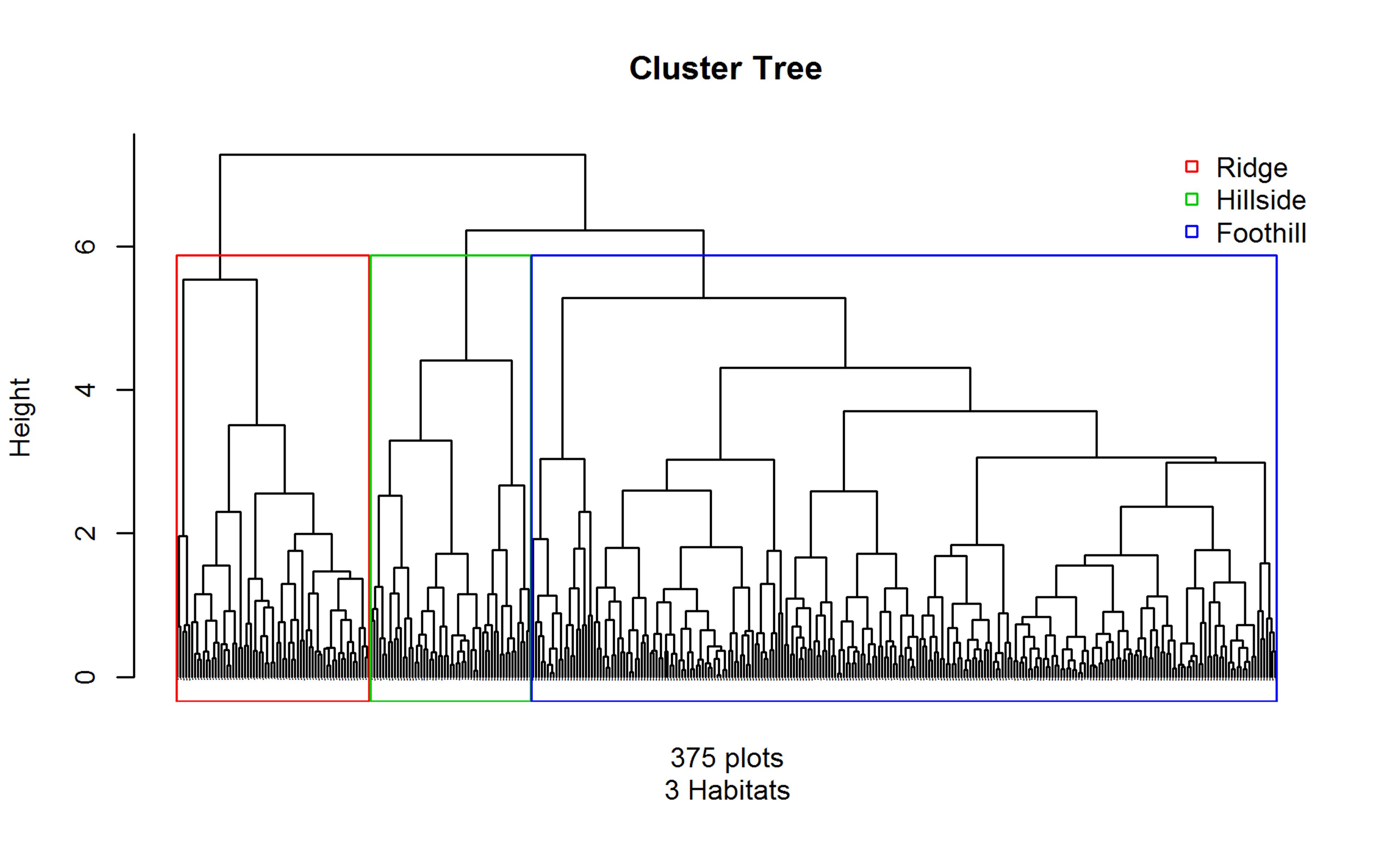

Supplement: Supplementary file 1 [file ECE3-8-11987-s001.jpg]

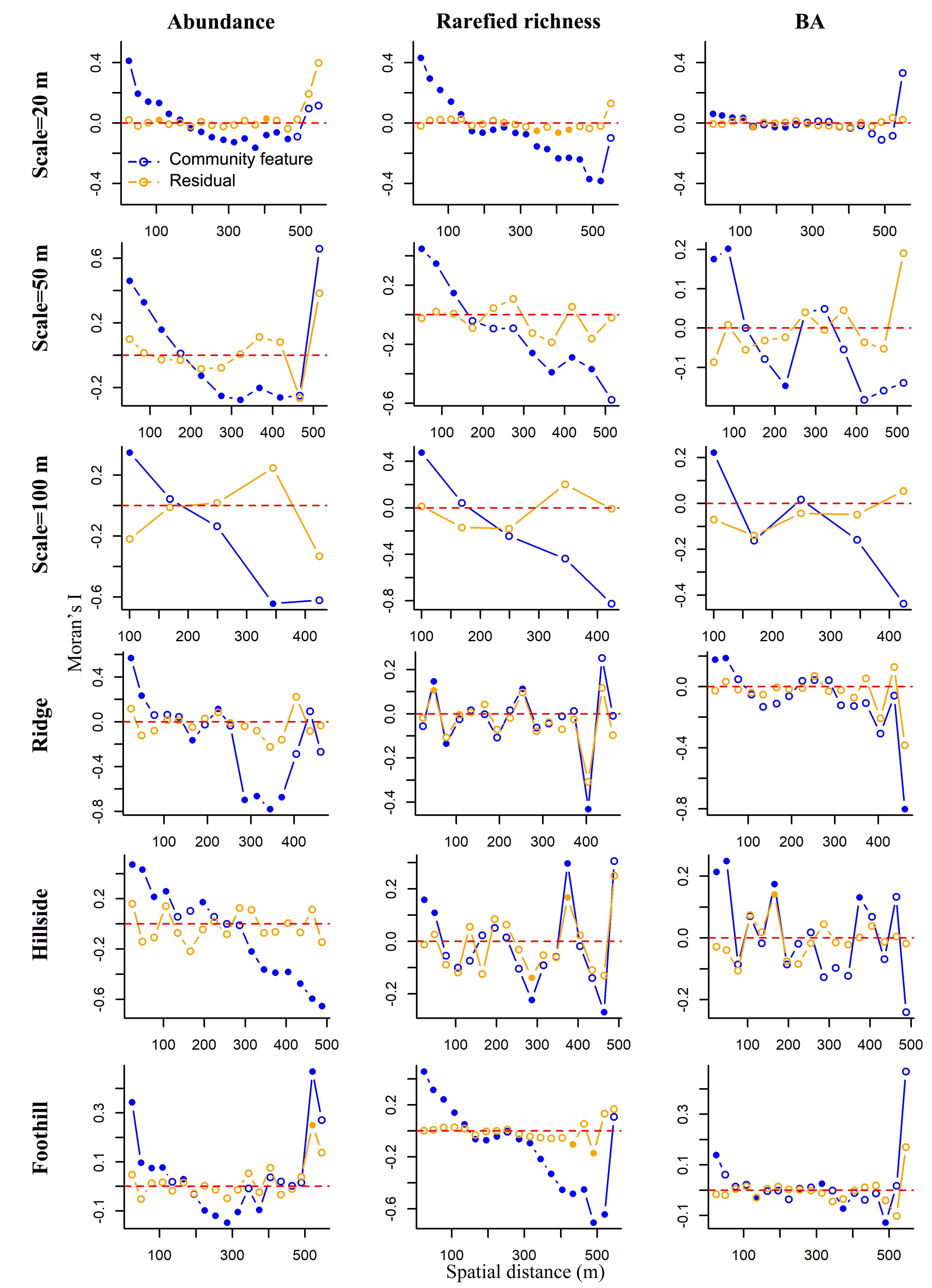

Supplement: Supplementary file 2 [file ECE3-8-11987-s002.jpg]

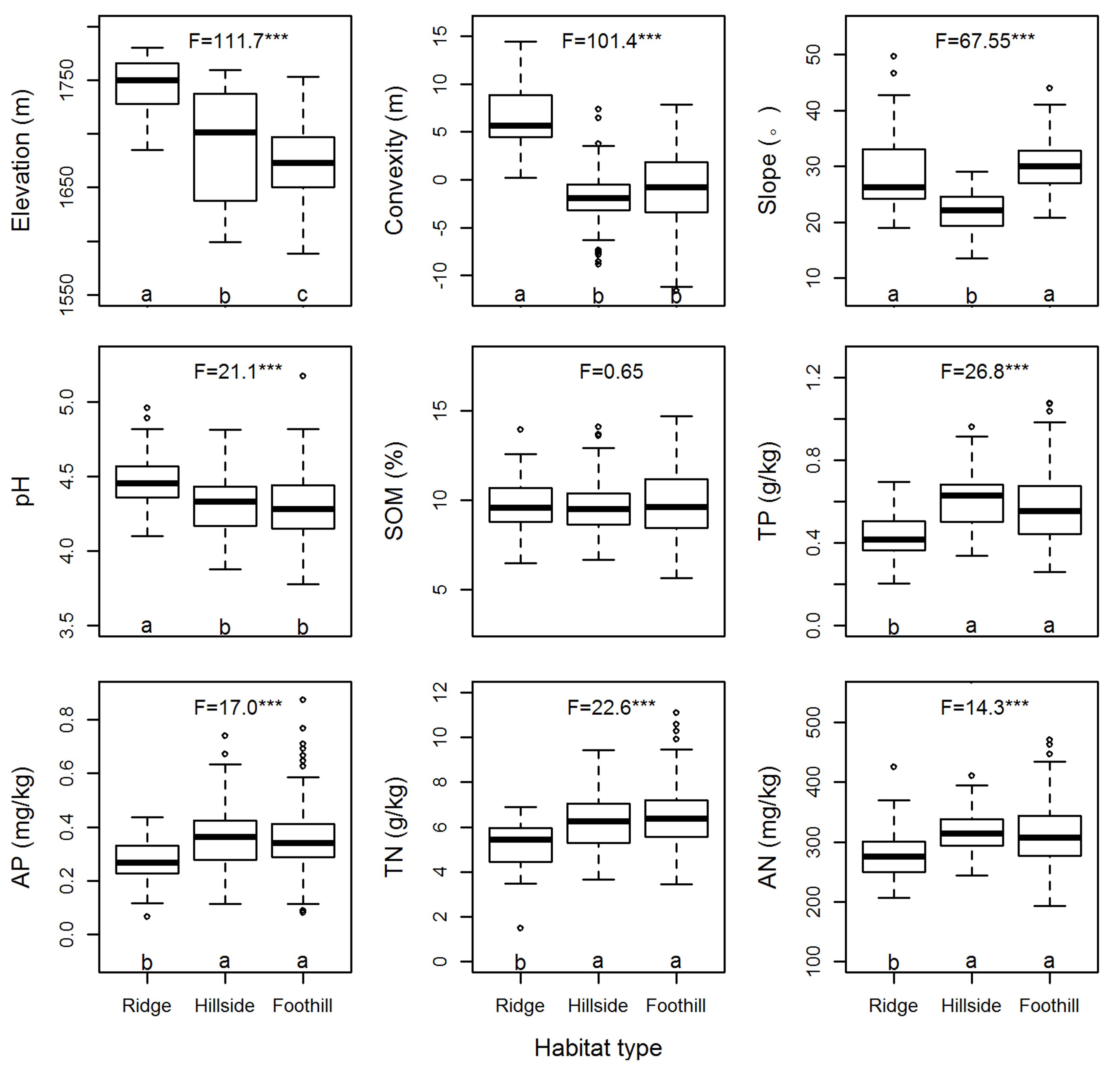

Supplement: Supplementary file 3 [file ECE3-8-11987-s003.jpg]

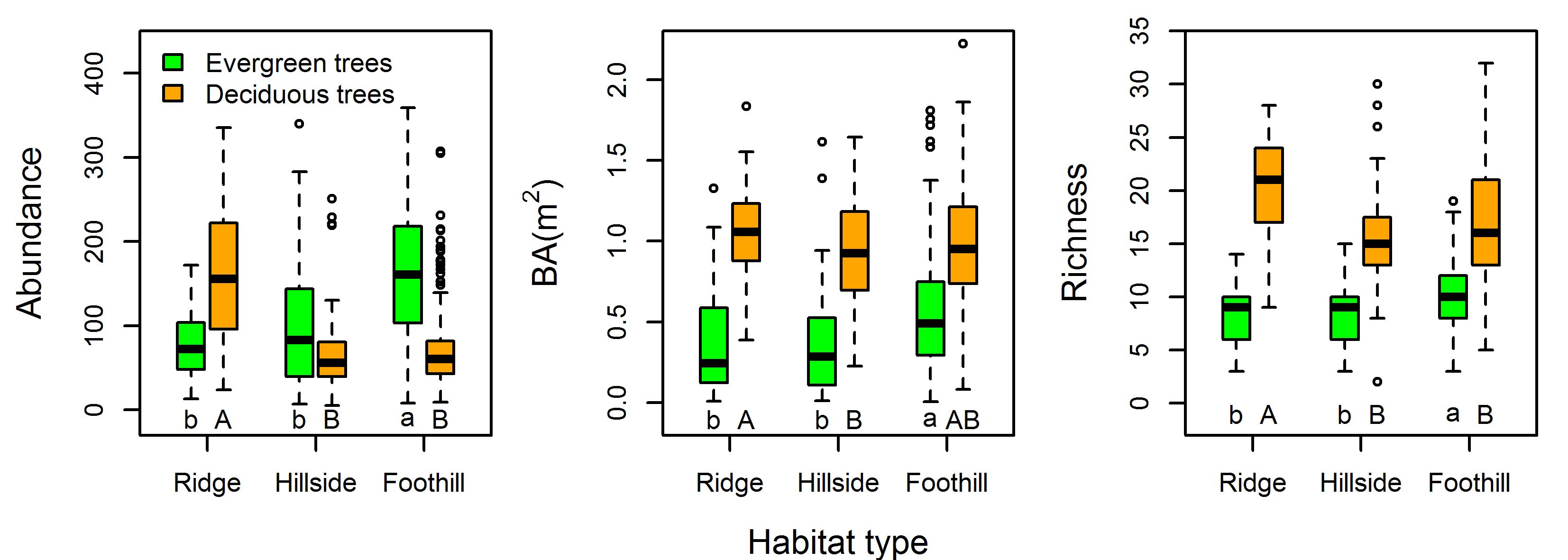

Supplement: Supplementary file 4 [file ECE3-8-11987-s004.jpg]

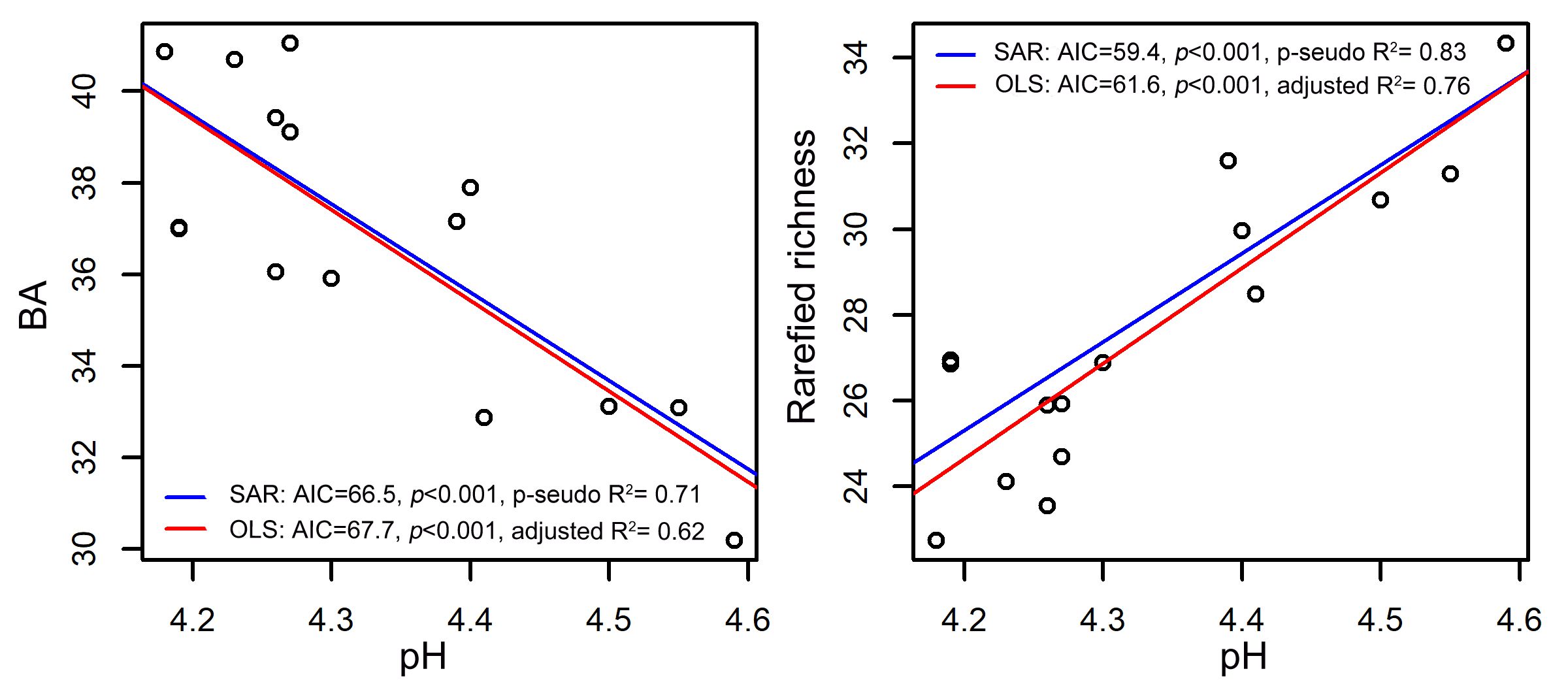

Supplement: Supplementary file 5 [file ECE3-8-11987-s005.jpg]

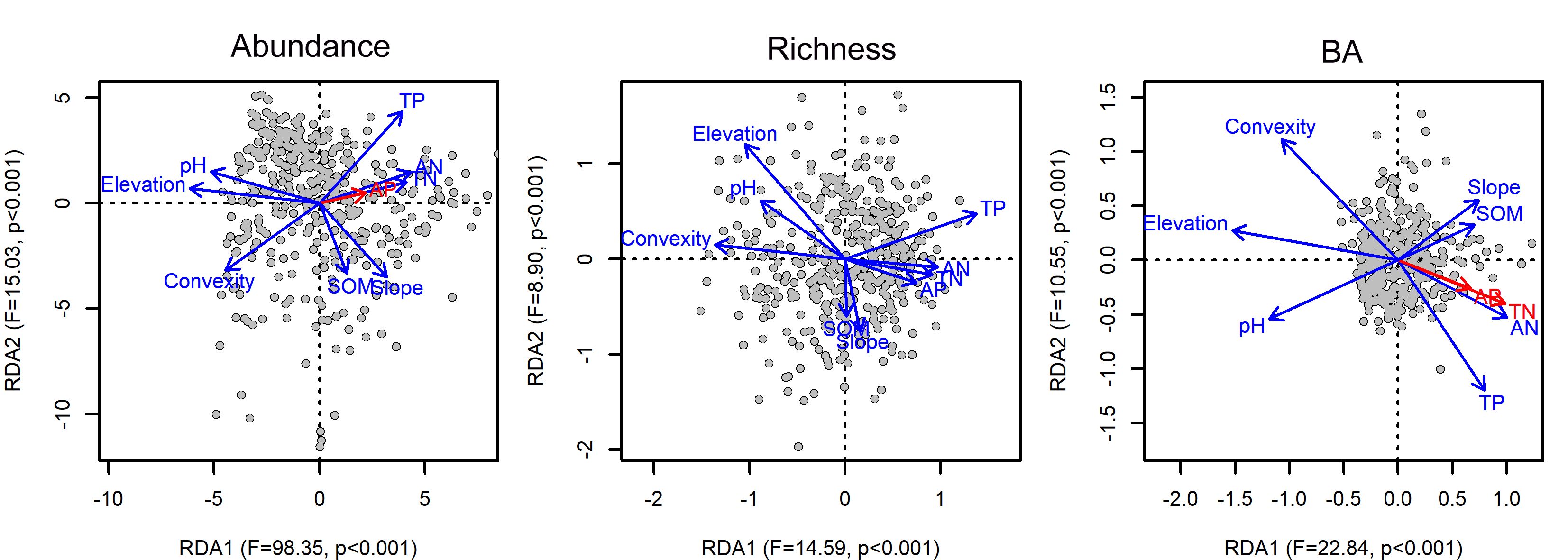

Supplement: Supplementary file 6 [file ECE3-8-11987-s006.jpg]
